# Patient-Reported Measures for Person-Centered Coordinated Care: A Comparative Domain Map and Web-Based Compendium for Supporting Policy Development and Implementation

**DOI:** 10.2196/jmir.7789

**Published:** 2018-02-14

**Authors:** Helen Lloyd, Hannah Wheat, Jane Horrell, Thavapriya Sugavanam, Benjamin Fosh, Jose M Valderas, James Close

**Affiliations:** ^1^ Community and Primary Care Research Group Plymouth University Peninsula Schools of Medicine & Dentistry Plymouth United Kingdom; ^2^ UK Centre for Tobacco and Alcohol Studies Nuffield Department of Primary Care Health Sciences University of Oxford Oxford United Kingdom; ^3^ Nuffield Department of Orthopaedics, Rheumatology & Musculoskeletal Sciences University of Oxford Oxford United Kingdom; ^4^ University of Exeter Medical School Exeter United Kingdom

**Keywords:** patient reported outcome measures, quality of life

## Abstract

**Background:**

Patient-reported measure (PRM) questionnaires were originally used in research to measure outcomes of intervention studies. They have now evolved into a diverse family of tools measuring a range of constructs including quality of life and experiences of care. Current health and social care policy increasingly advocates their use for embedding the patient voice into service redesign through new models of care such as person-centered coordinated care (P3C). If chosen carefully and used efficiently, these tools can help improve care delivery through a variety of novel ways, including system-level feedback for health care management and commissioning. Support and guidance on how to use these tools would be critical to achieve these goals.

**Objective:**

The objective of this study was to develop evidence-based guidance and support for the use of P3C-PRMs in health and social care policy through identification of PRMs that can be used to enhance the development of P3C, mapping P3C-PRMs against an existing model of domains of P3C, and integration and organization of the information in a user-friendly Web-based database.

**Methods:**

A pragmatic approach was used for the systematic identification of candidate P3C-PRMs, which aimed at balancing comprehensiveness and feasibility. This utilized a number of resources, including existing compendiums, peer-reviewed and gray literature (using a flexible search strategy), and stakeholder engagement (which included guidance for relevant clinical areas). A subset of those candidate measures (meeting prespecified eligibility criteria) was then mapped against a theoretical model of P3C, facilitating classification of the construct being measured and the subsequent generation of shortlists for generic P3C measures, specific aspects of P3C (eg, communication or decision making), and condition-specific measures (eg, diabetes, cancer) in priority areas, as highlighted by stakeholders.

**Results:**

In total, 328 P3C-PRMs were identified, which were used to populate a freely available Web-based database. Of these, 63 P3C-PRMs met the eligibility criteria for shortlisting and were classified according to their measurement constructs and mapped against the theoretical P3C model. We identified tools with the best coverage of P3C, thereby providing evidence of their content validity as outcome measures for new models of care. Transitions and medications were 2 areas currently poorly covered by existing measures. All the information is currently available at a user-friendly web-based portal (p3c.org.uk), which includes all relevant information on each measure, such as the constructs targeted and links to relevant literature, in addition to shortlists according to relevant constructs.

**Conclusions:**

A detailed compendium of P3C-PRMs has been developed using a pragmatic systematic approach supported by stakeholder engagement. Our user-friendly suite of tools is designed to act as a portal to the world of PRMs for P3C, and have utility for a broad audience, including (but not limited to) health care commissioners, managers, and researchers.

## Introduction

### Person-Centered Coordinated Care

Person-centered coordinated care (P3C) is at the nexus of 2 constructs: person-centered care [1] and care coordination [2]. It is a complex intervention with defined philosophical principles based on the individual’s right to self-determination [3-5] and collaborative approaches to care planning with patients [6]. Although it is perceived to be at the center of many new models of care in the United Kingdom, few (if any) services have a comprehensive understanding of what it is and how to implement it [7]. It can be defined as: “Care and support that is guided by and organised effectively around the needs and preferences of individuals.”

Five core domains of P3C have been previously identified: (1) information and communication, (2) care planning, (3) transitions, (4) patient-defined goals or outcomes, and (5) shared decision-making, as well as a number of further subdomains and component activities that are required for implementation [8]. It is a model that shares many similarities with the independent conclusions of others [9-11].

### Patient-Reported Measures

Patient-reported measures (PRMs) are questionnaires that probe individual patient perspectives on a range of health and health-related experiences and outcomes. A full list of PRMs covered by this study (including names and abbreviated names) is included in Multimedia Appendices 1 and 2.

### Patient-Reported Measures as Research Tools for Establishing Person-Centered Coordinated Care

The initial development of PRMs was largely driven by research [12], where patient-reported outcome measures (PROMs) were used to measure patient-reported symptoms, health status, functional status quality of life (QoL), or health-related quality of life in studies aiming at quantifying burden of disease on the impact of specific interventions. They are now also deployed in a variety of novel contexts, including clinical practice, quality improvement initiatives, and system-level feedback for health care management and commissioning [13]. This reflects an increasing emphasis from policy makers to utilize such measures for providing information about “how patients feel” [14] about the services they use and the interventions they are provided with.

Patient-reported experience measures (PREMs) are (in contrast to PROMs) tools for measuring patients’ experiences of care. Such measures have diversified into a large family of tools often covering core aspects of P3C, such as patient-practitioner communication, shared decision-making, and patient activation. We, thus, define a further category PRMs as *P3C-PRMS*, where aspects of P3C may be measured by experiences, processes, or health-related status.

Studies have established that person-centered approaches can reduce health care costs or lower use of health care services [15-17], with over half of P3C studies utilizing some form of PRM for evaluation [5,18-22]. Such findings have led to the increasing support for approaches such as self-management and patient activation in chronic conditions [23-25], which is one of the underpinning principles of chronic disease management in the United Kingdom and a continuing policy aim [26]. Such trends in policy and academic landscape are reflected internationally [27-29], including the US model of patient-centered medical homes [30].

### Patient-Reported Measures as Clinical Tools for Person-Centered Coordinated Care

More recently, PRMs (particularly PROMs) have become integrated into some areas of clinical practice [31]. In particular, oncology [32-34] and psychiatry [35,36] (with the advantages of a single clinical environment) have pioneered the use of PRMs for formalized feedback in routine clinical settings. A consistent finding is that PRMs can improve communication between the patient and the practitioner [32,33,37,38] and can result in a better quality and experience of care (particularly on processes) [34,37,39-44]. One systematic review reported that routine use of PROMs had an impact on the process of care in 65% of studies [44,45] and an impact on outcomes of care in 47% of studies [44,45].

### Patient-Reported Measures as System-Level Feedback for New Models of Care

PRMs are increasingly being utilized for system-level feedback for health care management, system auditing, and commissioning processes. This has largely been driven by the need for new service delivery models to cope with the demands of an ageing population with multiple long-term conditions (MLTCs), which has been called “the greatest challenge facing health systems around the world today” [21]. Patients with MLTCs may require an individualized approach to the delivery of health care as standard single disease guidelines and pathways appear ill-suited to improving relevant outcomes for this group [46-48]. PRMs provide a mechanism to systematically measure experiences and outcomes, which may be well aligned with the specific needs of these patients.

Such challenges are recognized by the policy landscape, which increasingly emphasizes person-centered approaches, often envisaged to be implemented in a systematic manner via the use of PRMs. For instance, in the United Kingdom, *Equity and Excellence: Liberating the NHS* [14] emphasized quality and patient involvement in decision making, committing to increase the use of PROMs. In 2014, the NHS (National Health Service) “five year forward view” highlighted how new models of care would become “more tailored to the individual,” and how “personalised care will only happen when statutory services recognise that patients’ own life goals are what count; that services need to support families, carers and communities; that promoting well-being and independence need to be the key outcomes of care; and that patients, their families and carers are often ‘experts by experience’.”

Such policy shifts have mirrored developments in the United States, where as early as 2001, the Institute of Medicine published its globally influential work *A New Health System for the 21st Century*, where one aim was a patient-centered system to drive forward improvements in health care quality [27]. More recently, Accountable Care Organizations (ACOs) [28,29] have been compared with the five-year forward view, and feedback of patient experience has been defined as one of the fundamental building blocks of high-performing primary care [49].

However, the use of PRMs for system-level aggregation is relatively new and faces novel challenges, with large-scale national surveys often being implemented to address the needs of policymakers— for example, particularly targeting accountability and transparency [50]. However, it has been argued that there is a “chasm” between the views of senior managers and clinicians at the front line [51]. Schemes have been criticized for survey length, being too generic and not focused at those who could most benefit from improvements in care, infrequent sampling frequency, slow feedback, and failure to use results to improve care [52], in addition to methodological problems and the difficulty of effectively using the data to actually improve care [52-55]. For example, the NHS Friends and Family Test (a single, global question of patient experience, essentially: “Would you recommend this service to friends and family?”) has been criticized as unsuitable as a comparator across organizations or as a basis for incentive payments [53]. The incentivization of PROMs for depression under the Quality and Outcomes Framework (a United Kingdom-based payment and performance management scheme for general practice) was quickly withdrawn after widespread criticism [55,56]. Other schemes—such as mandatory PROs for surgical procedures—have at least established that PRMs can be routinely collected and provide meaningful data [57-62].

At present, there is very little evidence that these tools can drive improvements in quality of care when used at a system level [34,63,64]. Despite the lack of evidence, health care is on a policy-driven trajectory that is placing an increasing emphasis on the use of PRM for system-level feedback. For instance, in the United Kingdom, this has been reflected in a number of national initiatives, where PRMs have been used as system-level monitoring tools in schemes such as the Vanguards and Better Care Funds (BCFs), often targeting patient groups such as older adults or those with long-term conditions (LTCs). However, many of these schemes are at early or pilot stages, and there is frequently a lack of agreement about what core outcome measures might be appropriate for new models of care. The use of these tools in novel contexts will require signposting, guidance, and clarity to a new range of stakeholders. This should include information on not only what existing measures are available but also associated information such as what they measure, references, the contexts in which they have been used, and the degree of psychometric validation.

The research reported in this paper was performed to address some of these shortcomings. We performed the following activities: (1) we constructed a user-friendly Web-based guidance portal (or “compendium”) of PRMS (p3c.org.uk); (2) next, for a targeted subset of these measures, we developed a “domain map” of measures that can be used to implement and measure P3C; (3) from this, we generated “short-lists” of measures according to specific categories; and (4) finally, we constructed an item-list from the mapped measures. Our user-friendly suite of tools is designed to act as a portal to the world of PRMs for P3C, and have utility for a broad audience, including (but not limited to) health care commissioners, managers, and researchers, thus allowing various stakeholders to rapidly identify measures that cover target domains of P3C.

## Methods

**Figure 1:**
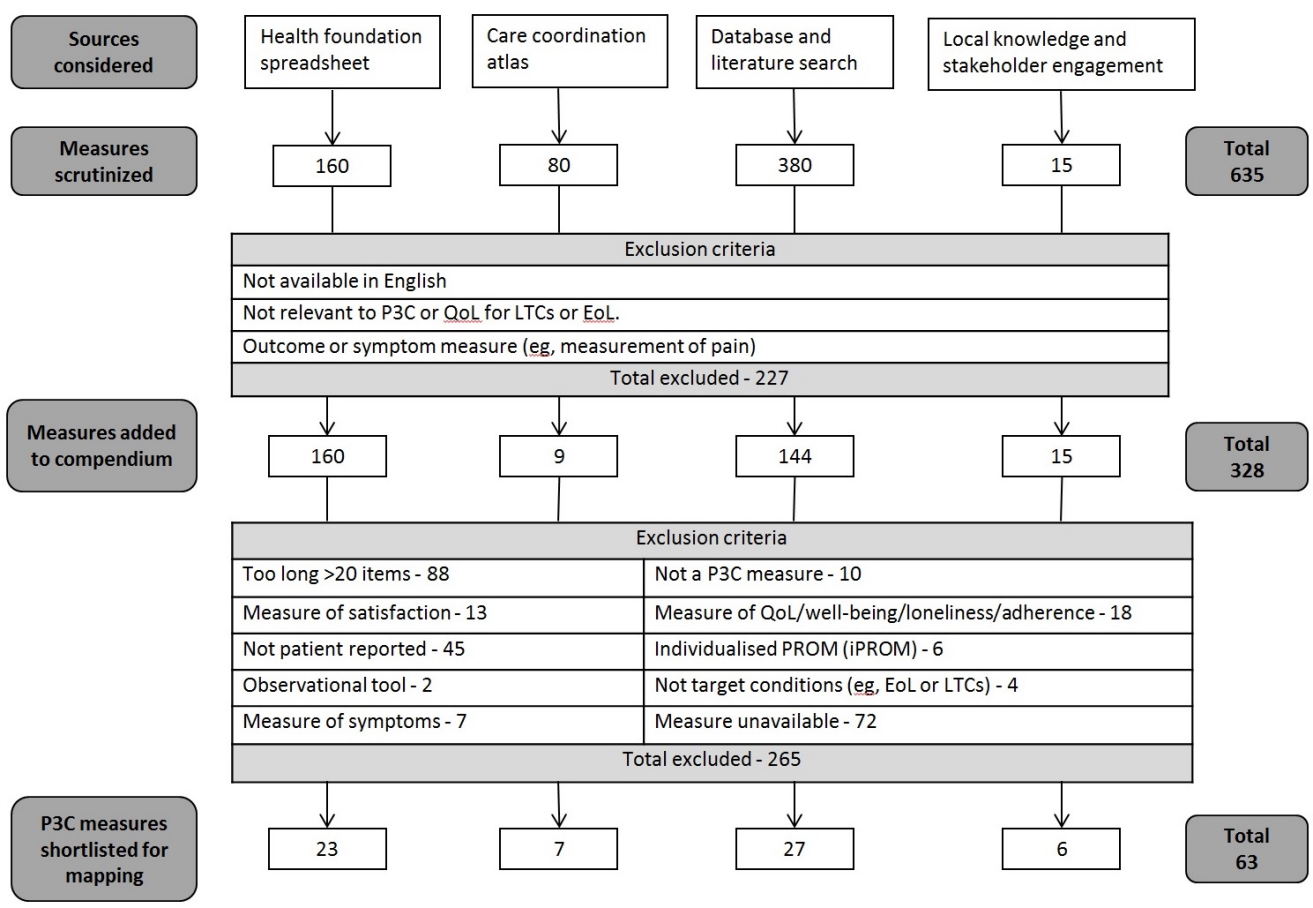
Overview of the identification, selection, and shortlisting process. P3C: Person-centered coordinated care; QoL: quality of life; LTC: long-term condition; EoL: end of life.

An overview of our identification, selection, and shortlisting process is presented in Figure 1 and explained in detail below.

### Identification of Relevant Measures

We considered 2 broad categories of measures for inclusion into the Web-based compendium. These were PRMs targeting P3C (P3C-PRMs) and QoL measures.

QoL measures were deemed a desirable addition to the dataset for the target audience of the compendium as QoL is often a target outcome of P3C interventions, many of the contexts in which P3C measures are used (eg, evaluation of P3C interventions) often also measure QoL [65], some studies have established correlations between person-centeredness and QoL [66], and policy guidance for person-centered interventions often includes QoL measures [67]. Furthermore, in the context of chronic illness where curative outcomes are not possible, there is an ethical imperative for health and social interventions to either maintain or improve people’s QoL.

As shown in Figure 1, the initial data source was the existing spreadsheet published in The Health Foundation’s document *Helping Measure Person-Centered Care* [65]. The subsequent sources represent new measures that were discovered *in addition* to The Health Foundation's comprehensive scan. See Multimedia Appendix 1 for full list of data sources and shortlisting criteria.

Due to the complexity and fragmentary nature of the existing data sources, complementary strategies were required and several data sources were interrogated to identify a broad list of candidate measures (see Figure 1). These sources were subjected to an initial “first screen,” and included if they were available in the English language and if they measured the construct of interest (P3C or QoL) for LTCs or end of life (EoL) (eg, first set of exclusion criteria in Figure 1). We rejected all non-native English measures, and our work makes the pragmatic assumption that all English-language instruments (eg, United States, United Kingdom, etc) are comparable (however, the compendium does retain information concerning country-of-origin). Measures that were health outcome or symptom measures (eg, measurement of pain) were also rejected. We examined the following sources.

#### Existing Compendiums and Lists of Measures

A recent study identified that there are more than 200 named and validated tools available for person-centered care alone [65], with a large number of tools that have only been validated in a single study. The Health Foundation’s evidence review, “helping measure person-centered care,” provided details of the most commonly researched person-centered measurement tools for person-centered care (see [65]). Given the availability of this resource, we initiated our search for P3C-PRMs with existing compendiums. Our initial list was seeded with 160 measures from The Health Foundation's list and 80 measures from the Care Coordination Atlas [68] (see Figure 1).

#### Literature Search

In contrast to P3C-PRMs (which often represent recent constructs developed under new models of care and are therefore often represented by a single, recent publication), many QoL measures (eg, EuroQoL-5D and short-form health survey) have often been subjected to rigorous validation in a large number of publications and subsequently subjected to head-to-head comparisons in a number of systematic reviews. We therefore used a review-of-reviews approach to identify QoL measures for the compendium (although these QoL measures were not included in the mapping procedure; see the section on short-listing of measures for mapping). These literature screens also identified several additional P3C-PRMs, and those that passed our screening criteria were added to our candidate list for domain mapping.

We limited our literature database search to Pubmed. Two groups of search terms were utilized—the first for QoL measures, and the second for P3C-related measures (Textbox 1). Due to the volume of literature, group 1 search term was limited to reviews over the last 2 years (as of March 2016). The search yielded 311 papers for review. Next, group 1 and group 2 search terms were combined for all papers (ie, not limited to reviews) for the last 2 years (as of March 2016). This produced 461 papers for review.

**Search terms used in the Pubmed database.**

Group 1 search terms (terms for measures): (PROM OR “Patient Reported Outcome“ OR PREM OR “Patient Reported Experience” OR PCOM OR “Patient Centered Outcome Measure” OR “Patient Centered Outcome Measures“)

Group 2 search terms (terms for P3C); an expanded list from [10]: (“Patient Centered” OR “Person Centered” OR “Patient Centered” OR “Person Centered” OR “Patient Co-Ordinated” OR “Patient coordinated” OR “Integrated care” OR “Shared decision” OR “Self Management” OR “Family centered” OR “Family centered” OR “Patient engagement” OR “Patient empowerment” OR “Patient activation” OR “Care Planning” OR “Goal Setting” OR “Client centered“ OR “Client centered” OR “Family centered” OR “Family centered” OR “Patient centric” OR “Patient centered” OR “Patient centered”)

We supplemented these literature searches with existing literature resources and databases that specifically comprise comparison of various PRMs:

1. “Consensus-based Standards for the selection of health Measurement Instruments” (COSMIN online database of references [69] (435 articles)
2. Papers that referenced “Terwee criteria” for measurement properties [70] (422 articles)
3. Papers that referenced EMPRO (evaluating measures of patient-reported outcomes), a tool for the standardized assessment of PROMs [71] (18 articles)
4. The Oxford PROMs group systematic reviews of PROMs for LTCs [72] (15 articles)

In total, 830 articles were screened from the literature searches, of which 130 were clearly identified (from the abstract) as covering measures with a relevant construct (eg, English-language QoL or P3C measures for LTCs and EoL). From these relevant articles, 380 measures were identified, of which 144 new measures fulfilled our inclusion criteria and were added to the compendium (236 rejected) (Figure 1).

#### Stakeholder Engagement

With the rapid, ongoing development of new models of care, we utilized local knowledge and relevant stakeholder engagement to identify further measures. A total of 15 further measures were identified in this manner.

Overall, a total of 328 relevant P3C-PRMs and QoL measures were identified from the above sources and added to the compendium). Please refer to Figure 1 for an overview of the data sources and shortlisting process and the spreadsheet in Multimedia Appendix 1 for full details of data sources and the shortlisting process. It should be noted that our website has additional measures beyond those included in this publication, for example, as it is not a static resource and also includes some measures represented with multiple versions.

### Short List of Person-Centered Coordinated Care-Patient-Reported Measure for Domain Map: Inclusion and Exclusion Criteria

After identification of P3C-PRMs and QoL measures with which to populate our compendium, we created a “map” of how items from the P3C-PRMs mapped to components of our model for P3C. We targeted measures that were concise and suitable for people with LTCs, MLTCs and for those at EoL.

As a first step, we applied a second set of inclusion and exclusion criteria to the identified PRMs in our compendium to produce a short list of P3C-PRMs. Our goal was to produce a list of P3C-PRMs that were succinct, did not measure patient satisfaction, and were suitable for use with people with LTCs or EoL. Our exclusion criteria to identify measures for the mapping exercise were as follows:

- *Short measures—* *less than 20 items (with some pragmatic exceptions):* We preferred short measures, as these are necessary to attain satisfactory response rates and reduce responder burden (especially with our specified target population of MLTCs and EoL), but also to ensure utility for routine health-service use.
- *Instruments were not measures of patient satisfaction*: Patient satisfaction is a widely criticized construct that is known to produce biased responses [73].
- *Patient-reported measures*: Measures that are patient-reported logically adhere to principles of patient-centered care. Furthermore, evidence also suggests that PRMs are more successful at predicting outcomes than either observations or physicians-reported measures [74,75]. However, in certain contexts (dementia; EoL), proxy measures (ie, measures completed by family member or professional) are unavoidable, and were therefore retained for shortlisting in these contexts.
- *Instruments were not measures of QoL, well-being, loneliness, or adherence:* Although our Web-based compendium does include QoL measures, these are not designed to measure aspects of P3C and cannot therefore be included in our mapping exercise.
- *Instruments were not individualized PROMs:* Individualized PROMs [76] such as the Person Generated Index [77] allow patients to modify the content or scoring system, prioritizing the symptoms to address [78]. Such patient empowerment is particularly salient to complex scenarios such as MLTCs [79]. However, this flexibility means that they cannot be mapped against domains of P3C.
- *Instruments were of utility for our disease-specific criteria (eg, LTC, EoL):* If instruments were specific to a condition that was not an LTC, they were excluded.
- *Measures were available to map*: Although we made best efforts to obtain copies of all target measures (eg, via references, Web searches and contacting authors), for many target measures, we could not obtain a copy of the measure, and they could not therefore be mapped.

Table 1 below describes the exclusion criteria used for the shortlisting process for P3C-PRMs. In total, 63 P3C-PRMs fulfilled our inclusion criteria and were shortlisted for the process of mapping (Figure 1; see Multimedia Appendix 1 for details).

**Table 1:** Exclusion criteria and number of measures rejected from mapping procedure. Patient-reported measures included in compendium (N=328) underwent second set of inclusion and exclusion criteria.

Exclusion criteria	Number excluded (n=265)
Too long; generally >20 items	88
Measure unavailable	72
Not patient reported	45
Measure of QoL^a^, well-being, loneliness, or adherence	18
Measure of satisfaction	13
Not a P3C^b^ measure	10
Measure of symptoms	7
Individualized patient-reported outcome measures^c^	6
Not target conditions (eg, EoL^d^ or LTCs^e^)	4
Observational tool	2

^a^QoL: quality of life.

^b^P3C: person-centered coordinated care

^c^Although “individualized patient-reported outcome measures” are inherently person-centered, domains are specified by patient and therefore cannot be mapped to the P3C model.

^d^EoL: end of life.

^e^LTCs: long-term conditions

### Domain Mapping of Measures for Person-Centered Coordinated Care

The selection criteria identified 63 candidate measures for P3C. Although standardized systems designed for head-to-head evaluation of instruments (such as EMPRO and COSMIN [69]) do exist, such methods are not appropriate in this context because (1) a wide diversity of instruments measure a range of overlapping constructs and (2) many instruments are only supported by a single validation paper.

As the aim of this work is to provide guidance to support the measurement and development of P3C through the use of PRMs, we instead assessed the shortlisted measures against a framework based on our model of P3C. This allowed us to construct a map of the questionnaires, allowing us to rapidly identify how various measures corresponded to components or constructs of person-centered approaches.

Our model of P3C was developed from our previous work including literature scoping and stakeholder engagement and contains all relevant domains of P3C [8]. It corresponds closely to well-accepted definitions of person-centered care such as the House of Care [11] and the National Voices “I” statements [9]. The model utilized in this paper includes the following primary domains (in addition to secondary domains; see Multimedia Appendix 2 for a full list of domains):

1. My goals and outcomes
2. Care planning
3. Transitions
4. Decision making
5. Information and Communication
6. Medication

Two researches (HW and JH) independently assigned each item on the questionnaire to domains that were derived from our model of P3C. Any inconsistencies in assignment between the 2 researchers were cross-checked and synchronized.

The final domain (medication) was not present within our previous model of P3C. However, it was represented with reasonable frequency across the items within the instruments that we mapped, and was therefore included as an additional domain for the purposes of this work. Mapping data for all instruments are provided in Multimedia Appendix 2.

## Results

### Domain Mapping of Person-Centered Coordinated Care Measures

**Figure 2:**
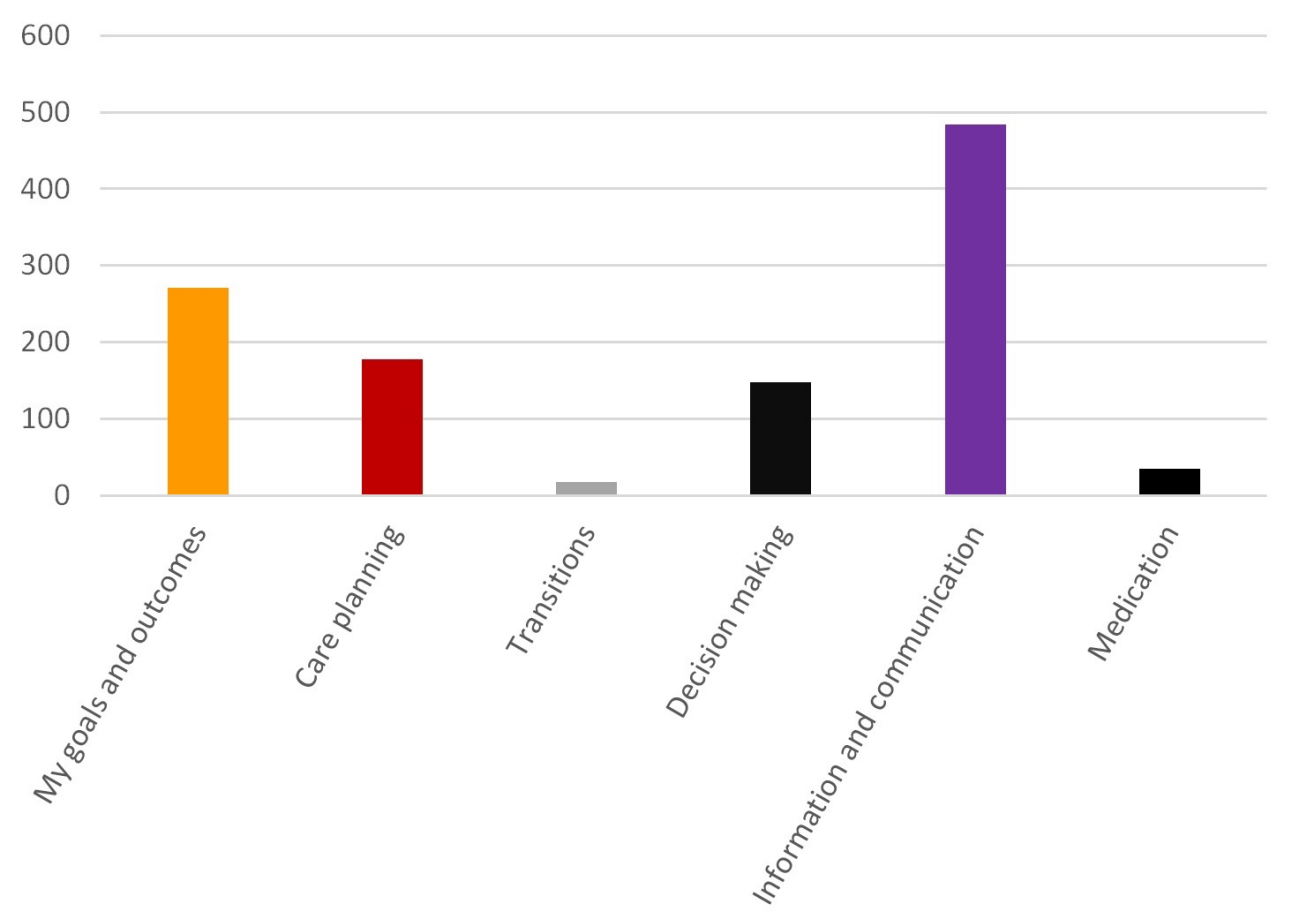
Number of items from all instruments mapping to specific domains of person-centered coordinated care (P3C). The x-axis is the total number of items (over all 63 mapped instruments) that map to a domain of P3C (y-axis).

In total, 855 items from the 63 shortlisted measures were mapped against our domain model of P3C. Multimedia Appendix 4 presents a summary of this domain mapping procedure, providing the number of items on each measure that map to specific domains of P3C. Multimedia Appendix 2 contains a full table of every item that maps to a domains or subdomain of P3C. The results are also graphically summarized in Figure 2.

Figure 2 and Multimedia Appendix 4 reveal that although Information and Communication (56.6%, 484/855) and Goals and Outcomes (31.69%, 271/855) are well covered by existing instruments, Care Planning (20.7%, 177/855) and Decision Making (17.2%, 147/855) are not as comprehensively covered. Only 17 items (2.0%, 17/855) were categorized as dealing with Transitions/Continuity of Care. However, transitions and care continuity have been recognized as a frequently problematic area of care coordination [9]. Future research and development of new (and existing) measures should be directed at addressing this limitation of existing instruments. Similarly, Medication (which has not been traditionally considered a domain of P3C) was only included in 35 items (4.1%, 35/855), with polypharmacy having been highlighted as a particular issue in the management of MLTCs [80].

Multimedia Appendix 4 displays the number of items that correspond to each domain (n) and the percentage (of the total) of items that map to this domain. These percentages are useful to identify the overall “balance” of a measure, that is, how heavily it corresponds to single or multiple domains.

### Pragmatic Shortlists

We also created shortlists of PRMs for P3C and QoL (available on our website p3c.org.uk/shortlist) to simplify, categorize, and signpost to the key entries in our dataset. These shortlists were developed via engagement with key stakeholders (NHS England, patients, commissioners, and professionals), where we defined 2 categories of shortlists: (1) according to domains of P3C, including measures that have good coverage of all domains, and (2) disease/age-specific categories (diabetes, cancer, psychiatry, stroke, heart failure, Parkinson’s, older people, dementia, and EoL).

These disease-specific categories include both the relevant measures of P3C for the specified condition and the measures of QoL that have been well-validated for the specified condition (see Methods section for the rationale for including QoL measures). The shortlisting of measures proceeded on the following principle: for QoL measures, there are generally a small number of well-used and validated measures (eg, EuroQoL-5D and short-form health survey), which are frequently included in systematic reviews. Therefore, these measures were selected on the basis of systematically reviewed psychometric properties (see references on website for details).

However, for measures of P3C, these were often newly developed or infrequently included within systematic reviews. Comparisons between the various measures are usually inappropriate due to the heterogeneous nature of the tools, and there is insufficient evidence to recommend one survey tool over another [65]. Thus, we instead shortlisted based on a range of pragmatic criteria. We utilized our domain map to identify measures that covered each of the 6 domains of P3C. We also preferred measures that had reasonable psychometric properties, had been codesigned with patients, and had been developed according to recent constructs of P3C. Finally, we also took into account the context (hospital, primary care, nursing home or rehabilitation); whether patients, staff, or both are the target; the preferred length or number of survey items; and whether the focus is on the broad concept of P3C or a narrower subcomponent (such as communication or shared decision-making) [65]. These criteria allowed us to assign measures according to domains of P3C: generic measure; measures for goals and outcomes; care planning; transitions; decision making; and information and communication.

**Figure 3:**
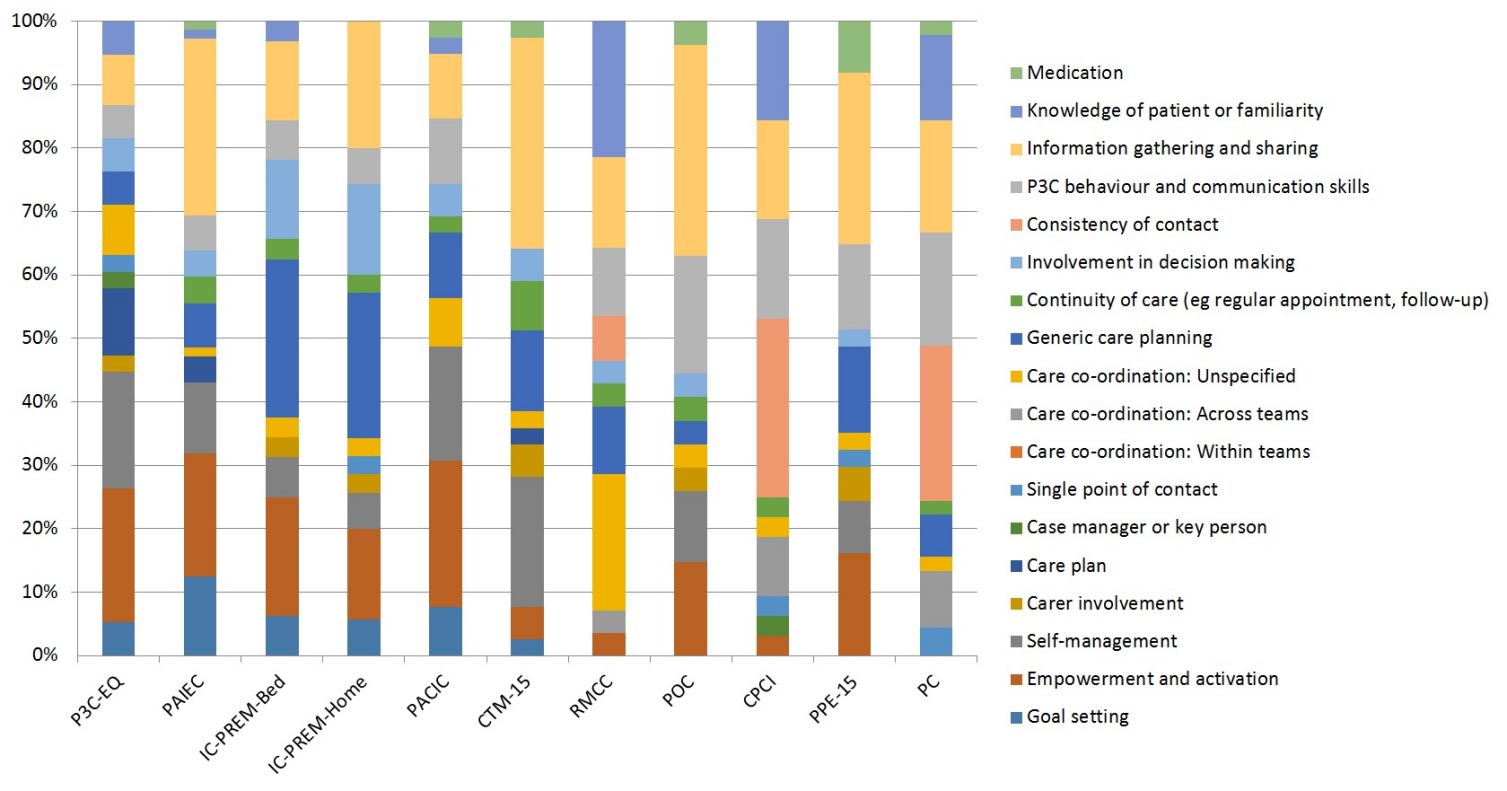
Example domain map for 11 measures with broad domain coverage of person-centered coordinated care (P3C) (mapping to ten or more different subdomains of P3C), in order from the best coverage (on the left) to less broad coverage (on the right). P3C-EQ: person-centered coordinated experience questionnaire; PAIEC: patient assessment of integrated elderly care; IC-PREM-Bed: integrated care patient-reported measure, bed-based version; IC-PREM-Home: integrated care patient-reported measure, home-based version; PACIC: patient assessment of care for chronic conditions; CTM-15: care transitions measure, 15 item version; RMCC: relational and management continuity of care; POC: perceptions of care; CPCI: components of Primary Care Index; PPE-15: Picker Patient Experience Questionnaire; PC: patient perception of continuity instrument.

### Measures With Broad Coverage of All Person-Centered Coordinated Care Domains

To identify measures with broad coverage of P3C, we identified the number of different subdomains of P3C that each measure mapped to (see Multimedia Appendix 2 and Multimedia Appendix 4 for details). In total, 11 measures mapped to at least 10 different subdomains of P3C of a possible 18, according to the criteria used in this publication (see Figure 3 and Multimedia Appendix 2 for full details). These were as follows: (1) the Person-Centered Coordinated Care Experience Questionnaire (P3C-EQ; 11 items; covers 13 subdomains of P3C), a recently developed measure designed to provide extensive coverage of the domains of P3C [81]; (2) the Patient Assessment of Integrated Elderly Care (PAIEC; 21 items; covers 12 subdomains of P3C), a recently modified version of the Patient Assessment of Care for Chronic Conditions (PACIC) specifically designed for older populations [82]; and (3) and (4) IC-PREM-Home and IC-PREM-Bed (both 15 items; covers 11 subdomains of P3C) are a pair of PREMs that have been designed specifically to evaluate the delivery of person-centered care for older people in intermediate care services [83]; (5) the Patient Assessment of Care for Chronic Conditions (PACIC; 20 items; covers 11 subdomains of P3C), a well-established tool for measuring patient experience of chronic illness care that is applicable in many settings [16]; (6) the Care Transition Measure (CTM-15; 15 items; covers 11 subdomains of P3C), cited as being the most widely used measure of care transition quality [84]; (7) the Relational and Management Continuity of Care (RMCC; 25 items; 10 subdomains of P3C) [85]; (8) Perceptions of care (POC; 21 items; 10 subdomains of P3C); (9) Components of Primary Care Index (CPCI; 20 items; 10 domains of P3C) [86]; (10) the Picker Patient Experience Questionnaire (PPE-15; 15 items; 10 subdomains of P3C), originally designed for use in inpatient care settings [87]; (11) and the Patient Perception of Continuity Instrument (PC; 23 items; 10 subdomains of P3C).

### List of Person-Centered Coordinated Care Items and Questions

Our mapping exercise also facilitated the construction of database of specific questions (items) for P3C. This allows the user to search according to a domain (or subdomain) of P3C, and return all relevant items, including associated information such as originating instrument; type of scale; response options; and links to the measure and relevant page on the p3c.org.uk website. The item database can be interrogated via a spreadsheet contained in Multimedia Appendix 3. It is intended as a tool of particular utility in the identification of measures that correspond to specific domains, in addition to having utility in the generation of new measures.

### Web-Based Database of Measures for Person-Centered Coordinated Care

**Figure 4:**
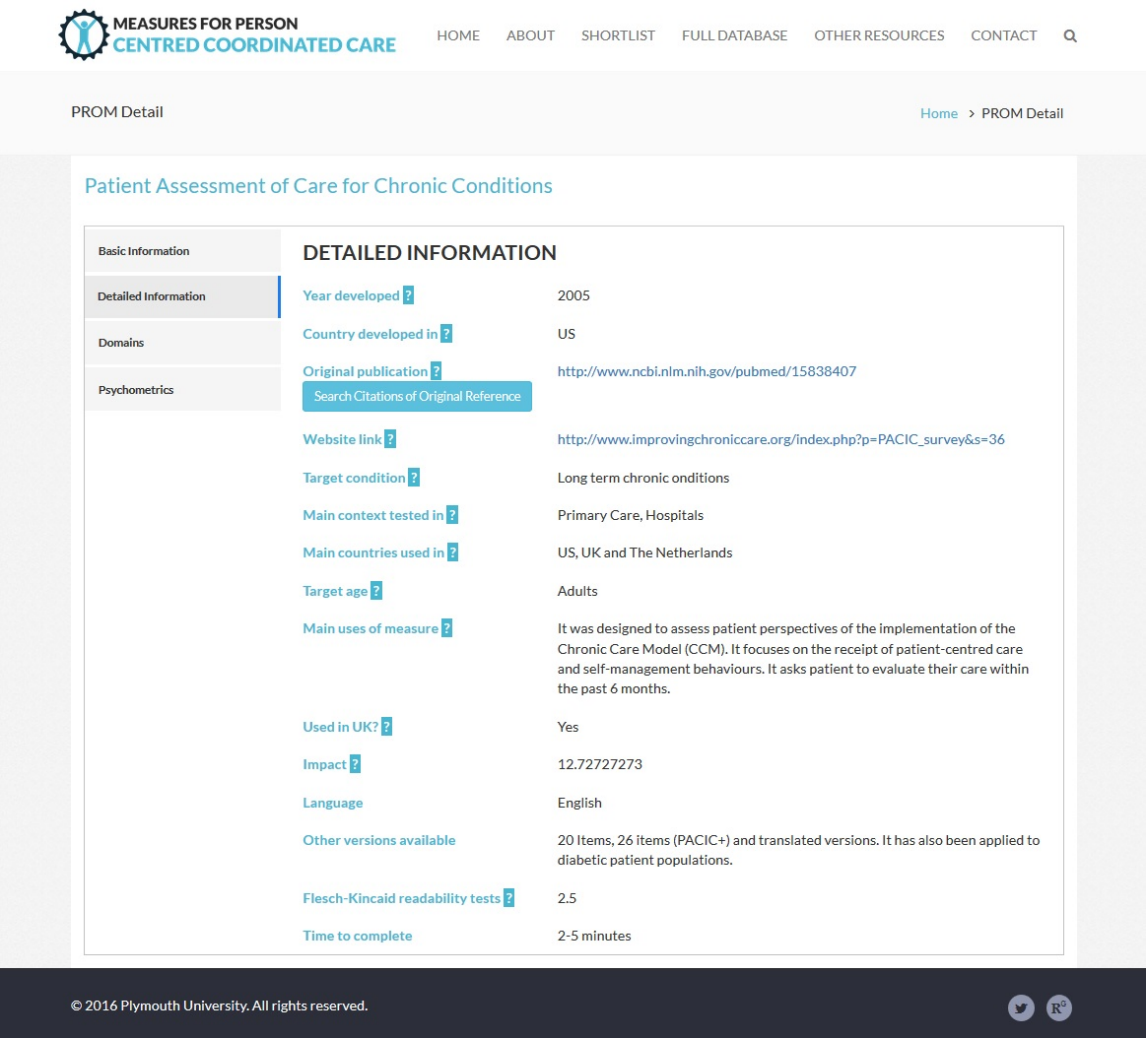
Example entry from website.

**Figure 5:**
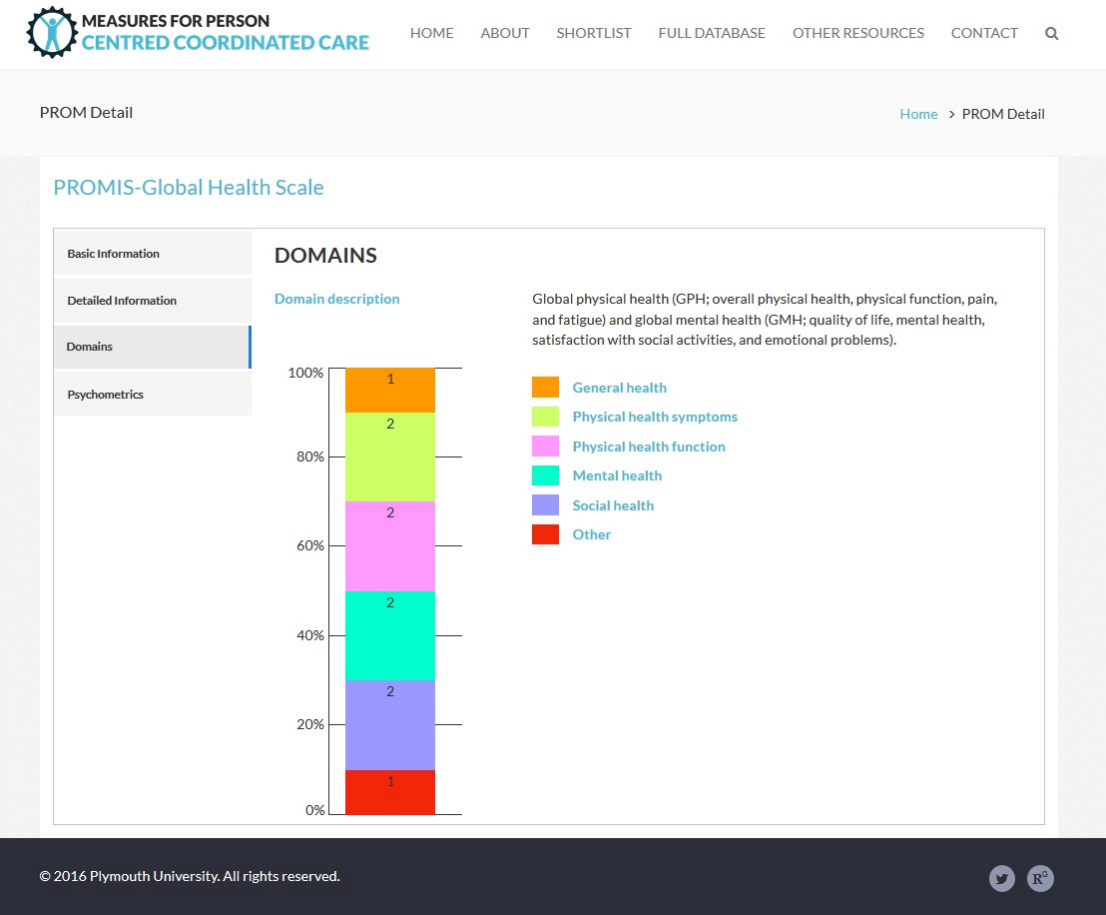
Example entry from website, showing domain mapping information.

We utilized our assembled database of measures to compile a free Web-based repository of measures for P3C, which includes both P3C-PRMs and QoL measures. All our data have been made publicly available, and include many more measures in addition to the list previously published by The Health Foundation [65]. Although the database of measures used for this paper (Multimedia Appendix 1) represents a static snapshot of measures (as of June 2016), the Web-based database is a flexible, extensible, and updated product, and includes a large number of instruments (333 at time of publication), many of which are not included in this paper (including multiple versions of instruments when these are available). In addition, it includes a wealth of supporting information (see Figures 4 and 5 for an example entry from the website). The supporting information includes the following details:

1. Basic information (name; abbreviated name; description; type of measure; respondent; licensing information and link; and link to the questionnaire).
2. Detailed information (year developed; country developed; link to original validation paper; a search tool for automated literature searches; and target conditions and age). It is worth noting that our database includes measures that originate from various international contexts (provided in the detailed information), and that validation or adaptation of measures may be required before they are deployed in novel contexts.
3. Domains of P3C or QoL that the instrument covers.
4. Psychometrics—either a description of psychometric properties or (where available) a graphical indication of the results of systematic review of the psychometric properties of the instrument.

Because the full database was drawn from a wide diversity of sources, the Web-based database is uneven in the level of detail about PRMs. Generally, those that are well-validated measures have more complete information available.

## Discussion

### Principal Findings

PRMs are evolving into an increasingly diverse suite of tools that are now being utilized within a diversity of novel contexts, where it has recently been argued that “providers need more support and guidance” [88]. In response to such calls, the primary output of this work was a systematic characterization of PRMs with utility in the evaluation and delivery of P3C, including an Web-based portal of measures; a domain “map” of 63 shortlisted measures for P3C care, allowing for rapid identification of measures that cover target domains; generation of “short-lists” of measures according to specific categories; and the construction of an item-list from the mapped measures.

For such PRMs to be used to drive improvements in health or health care services, the logic of the intervention should be well understood, it should be codesigned with all relevant parties, and mechanisms of feedback should be appropriately designed to drive the desired change (while also avoiding undesired effects such as “tunnel vision”) [88]. In the area of person-centered care, such arguments may be particularly pertinent, where practitioners often mistakenly believe they are delivering person-centered care when they are not [89]. In this regard, our tools are accompanied by a detailed guidance document, which support the use of metrics to drive improvements [8]. This provides a detailed definition of P3C and guidance for implementation and development using quality improvement strategies with embedded metrics. It demonstrates how principles of person-centered care can be translated into actions through 4 core practice routines [3]: (1) establish the partnership by eliciting the a narrative, (2) agree and formulate a plan based on shared decision-making, (3) safeguard the plan in a document, and (4) coordinate the plan.

Linking process and outcomes of P3C to the 4 core practice routines described above provides a way in which to measure and support ongoing development of P3C by using measures that probe these areas. The compendium and comparative mapping work makes this linking possible. Furthermore, understanding the processes and outcomes in relation to these core practices will allow for the development of training and improvement strategies tailored to this framework, thus allowing practitioners to develop their understanding of P3C and focus on improving particular aspects of their practice. Each practice routine is important for this outcome, and this could be measured from the perspective of the patient using a number of generic tools (eg, person-centered coordinated experience questionnaire [P3CEQ], patient assessment of integrated elderly care [PAIEC], Patient Assessment of Care for Chronic Conditions [PACIC], Care Transitions Measure [CTM-15], etc) or instruments for a specific task (eg, instruments for shared decision-making such as CollaboRATE and SURE (Sure of myself, Understand information, Risk-benefit ratio, Encouragement).

### Conclusions

Our tools are designed to help address a number of the above issues and have utility for a range of stakeholders. In particular, the Web-based database and pragmatic shortlists are designed to be a user-friendly front end that simplifies and signposts to a vast and complex literature. These tools act as a portal for health care professionals that may not have academic knowledge of PRMs (eg, health care managers and commissioners). Our tools enable rapid identification of suitable PRMs (eg, for a specific disease or concept), and thereby can help avoid the unnecessary development of new measures when suitable tools already exist. Furthermore, the website is an extensible, ongoing product and includes further measures and information beyond the context of this publication. Used along with our detailed guidance for commissioners [8], collectively these tools support the implementation and development of P3C. Furthermore, the tools are also designed to be of utility to researchers, with the Web-based database including features such as automated literature searches.

The domain map (Multimedia Appendix 2) establishes content validity for a number of instruments designed under new models of care (eg, P3C-EQ, IC-PREM-Bed, IC-PREM-Home and CTM-15), revealing that they do indeed cover more domains of P3C than other measures (as defined by our model, which is closely related to others) [9,11]. In fact, our recently developed measure, the P3C-EQ [81], has a broader coverage of aspects of P3C than any other measure we identified. Furthermore, the mapping exercise highlights potential shortfalls in the coverage of measures (especially transitions and care continuity). PRMs for holistic measurement for P3C are a relatively new concept, and the item map thus highlights how the future development and adaptation of measures could proceed (ie, *transitions and medication* and *polypharmacy* are currently underrepresented). Finally, the item database itself is a useful resource to aid in this ongoing development of improved measures.

## Appendix

Multimedia Appendix 1 Full list of data sources and shortlisting process.

Multimedia Appendix 2 Full mapping details.

Multimedia Appendix 3 Item list/database.

Multimedia Appendix 4 Results of mapping exercise for 63 person-centered coordinated care patient-reported measures.

## Abbreviations

COSMIN: consensus-based standards for the selection of health measurement instruments
CTM-15: care transitions measure, 15-item version
EMPRO: evaluating measures of patient-reported outcomes
EoL: end of life
LTC: long-term condition
MLTCs: multiple long-term conditions
P3C: person-centered coordinated care
P3C-EQ: person-centered coordinated experience questionnaire
P3C-PRM: person-centered coordinated care patient-reported measure
PACIC: patient assessment of care for chronic conditions
PAIEC: patient assessment of integrated elderly care
PREM: patient-reported experience measure
PRM: patient-reported measure (an umbrella term we utilize for all measures)
PROM: patient-reported outcome measure
QoL: quality of life
