# Supplementary material for: Patient-Reported Measures for Person-Centered Coordinated Care: A Comparative Domain Map and Web-Based Compendium for Supporting Policy Development and Implementation
Source: J Med Internet Res. 2018 Feb 14;20(2):e54. doi: 10.2196/jmir.7789 (PMC5830608; doi:10.2196/jmir.7789)
Supplement: Multimedia Appendix 4 [file jmir_v20i2e54_app4.pdf]

Multimedia Appendix 4. Results of mapping exercise for 63 person-centered coordinated care patient-reported measures. Displays the number of items that correspond to each domain (n) and the percentage (of the total) of items that map to this domain. These percentages are useful to identify the overall "balance" of a measure, that is, how heavily it corresponds to single or multiple domains. Due to space constraints, the names of the measures have been abbreviated; the full names of the measures can be found at the bottom of the table. Note that a single measure can map to multiple domains, so the total of the numbers mapping to each domain for an instrument is usually higher than the number of items (final column). Due to this, percentages are calculated as the percentage of the sum of the items mapped to domains (eg, rather than the number of the items in the final column). This means that the percentages across domains always add up to 100%, making it easier to identify the balance of each measure. The final column displays the number of different subdomains of P3C that the instrument maps to (see Multimedia Appendix 2), with a higher number indicating a better coverage of various aspects of P3C.

Name of measure	Goals and outcomes	Care planning	Transitions	Decision making	Information and Communication	Medication	Number of items	Number of subdomains
	n (%)	n (%)	n (%)	n (%)	n (%)	n (%)		
CARE[a]	6 (38)	1 (6)	0 (0)	1 (6)	8 (50)	0 (0)	10	7
CTM-15[b]	12 (34)	5 (14)	3 (9)	2 (6)	12 (34)	1 (3)	15	11
CollaboRATE	3 (33)	3 (33)	0 (0)	1 (11)	2 (22)	0 (0)	3	6
CAT[c]	2 (12)	0 (0)	0 (0)	1 (6)	14 (82)	0 (0)	15	4
CCM[d]	12 (32)	3 (8)	0 (0)	5 (14)	17 (46)	0 (0)	21	8
DISQ[e]	0 (0)	0 (0)	0 (0)	0 (0)	8 (100)	0 (0)	12	2
ESQ[f]	0 (0)	1 (17)	0 (0)	0 (0)	5 (83)	0 (0)	12	3
HCCQ[g]	1 (8)	0 (0)	0 (0)	0 (0)	11 (92)	0 (0)	13	3
HowRwe	0 (0)	0 (0)	0 (0)	0 (0)	2 (100)	0 (0)	4	2
IAPT-PbR	4 (33)	0 (0)	0 (0)	4 (33)	4 (33)	0 (0)	9	4
IDPCS[h]	6 (23)	1 (4)	0 (0)	3 (12)	15 (58)	1 (4)	19	6
IntegRATE	0 (0)	4 (37)	0 (0)	0 (0)	2 (33)	0 (0)	4	4
JSPPPE[i]	0 (0)	0 (0)	0 (0)	0 (0)	5 (100)	0 (0)	5	2
LTCQ-6[j]	6 (55)	2 (18)	0 (0)	1 (9)	2 (18)	0 (0)	6	5
MPI[k]	6 (23)	2 (8)	0 (0)	3 (12)	14 (54)	1 (4)	19	6
NORPEQ[l]	0 (0)	0 (0)	0 (0)	0 (0)	3 (100	0 (0)	8	1
Dyadic OPTION	6 (26)	0 (0)	0 (0)	12 (52)	5 (22)	0 (0)	12	4
OPPQNCS[m]	1 (14)	3 (43)	1 (14)	1 (14)	1 (14)	0 (0)	19	9
PACIC[n]	11 (38)	7 (24)	1 (3)	2 (7)	7 (24)	1 (3)	20	11
P-CIS[o]	6 (67)	0 (0)	0 (0)	2 (22)	1 (11)	1 (11)	20	4
I-PAHC[p]	1 (6)	0 (0)	0 (0)	0 (0)	9 (56)	6 (38)	25	4
PEQ[q](Steine)	3 (38)	2 (25)	0 (0)	2 (25)	1 (13)	0 (0)	15	6
PFC[r]	2 (9)	6 (27)	0 (0)	2 (9)	12 (55)	0 (0)	16	7
PPRQ[s]	12 (34)	1 (3)	0 (0)	5 (14)	17 (49)	0 (0)	23	9
M-PICS[t]	1 (4)	5 (19)	0 (0)	5 (23)	14 (54)	0 (0)	20	5
PCCQ[u]	1 (25)	0 (0)	0 (0)	0 (0)	3 (75)	0 (0)	17	3
PPE-15[v]	8 (26)	7 (23)	0 (0)	1 (3)	12 (39)	3 (10)	15	10
TES[w]	1 (3)	8 (22)	0 (0)	10 (28)	7 (19)	10 (28)	10	6
IC-PREM-Home[x]	6 (20)	9 (30)	1 (30)	5 (17)	9 (30)	0 (0)	15	11
IC-PREM-Bed[y]	6 (22)	9 (33)	1 (4)	5 (15)	7 (26)	0 (0)	15	11
4HPQ[z]	4 (15)	6 (23)	1 (4)	1 (4)	14 (54)	0 (0)	15	9
PAIEC[aa]	14 (33)	9 (21)	3 (7)	3 (7)	13 (30)	1 (2)	21	12
QPP-SF[ab]	4 (15)	2 (7)	0 (0)	1 (4)	19 (70)	1 (4)	24	9
QOC-10[ac]	1 (20)	2 (40)	0 (0)	1 (20)	1 (20	0 (0)	10	5
RMCC[ad]	1 (4)	7 (30)	1 (4)	1 (4)	13 (57)	0 (0)	25	10
SES6G[ae]	6 (100)	0 (0)	0 (0)	0 (0)	0 (0)	0 (0)	6	2

SRS[af]	2 (40)	0 (0)	0 (0)	0 (0)	3 (60)	0 (0)	4	4
SDM-Q-9[ag]	5 (29)	1 (6)	0 (0)	6 (35)	5 (29)	0 (0)	9	4
STAR-P[ah]	4 (19)	4 (19)	0 (0)	2 (10)	11 (52)	0 (0)	12	7
SURE scale	2 (22)	0 (0)	0 (0)	4 (44)	3 (33)	0 (0)	4	3
VOICE[ai]	2 (13)	1 (6)	0 (0)	1 (6)	9 (56)	3 (19)	19	8
Visual CARE measure (adult)	7 (37)	2 (11	0 (0)	1 (5)	9 (47)	0 (0)	5	6
Visual CARE measure (child)	3 (30	1 (10)	0 (0)	1 (10)	5 (50)	0 (0)	10	6
QOC-revised[aj]	5 (17)	6 (20)	0 (0)	2 (7)	17 (57)	0 (0)	19	7
CES-P[ak]	2 (20)	2 (20)	0 (0)	1 (10)	5 (50)	0 (0)	23	9
CPCI[al]	1 (4)	5 (21)	1 (4)	0 (0)	17 (71)	0 (0)	20	10
DSES[am]	7 (35)	3 (15)	0 (0)	9 (45)	1 (5)	0 (0)	11	5
DCS[an]	8 (23)	0 (0)	0 (0)	16 (46)	11 (31)	0 (0)	16	5
PAV-COM[ao]	7 (41)	1 (6)	1 (6)	1 (6)	7 (41)	0 (0)	9	6
PC[ap]	0 (0)	7 (23)	1 (3)	0 (0)	21 (70)	1 (3)	23	10
CPS[aq]	1 (33)	0 (0)	0 (0)	1 (33)	1 (33)	0 (0)	1	3
HCS[ar]	4 (50)	2 (25)	0 (0)	1 (13)	1 (13)	0 (0)	4	6
PAM-13[as]	13 (68)	0 (0)	0 (0)	0 (0)	5 (26)	1 (5)	13	4
P3CEQ[at]	1 (6)	8 (47)	0 (0)	2 (12)	6 (35)	0 (0)	11	13
OEQ[au]	2 (29)	1 (14)	0 (0)	1 (14)	3 (43)	0 (0)	11	6
POC[av]	5 (23)	2 (9)	1 (5)	1 (5)	12 (55)	1 (5)	21	10
POS[aw]	2 (50)	1 (25)	0 (0)	0 (0)	1 (25)	0 (0)	12	3
PEQ[ax] (NHS)	5 (36)	2 (14)	0 (0)	1 (7)	6 (43)	0 (0)	19	9
OxPIE[ay]	5 (26)	3 (16)	1 (5)	2 (11)	8 (42)	0 (0)	11	9
QEOLC-10[az]	2 (15)	1 (8)	0 (0)	1 (8)	9 (69)	0 (0)	10	7
HCSHS[ba]	3 (13)	4 (17)	0 (0)	0 (0)	12 (52)	4 (17)	16	7
HCEQ[bb]	9 (43)	3 (14)	0 (0)	3 (14)	9 (29)	0 (0)	10	5
CCCQ[bc]	11 (32)	12 (35)	0 (0)	10 (29)	1 (3)	0 (0)	14	6
Total	271 (32)	177 (21)	17 (2)	147(17)	454 (57)	35 (4)	855	

[a]Consultation and Relational Empathy Scale (CARE), [b]Care Transitions Measure (CTM-15), [c]Communication Assessment Tool (CAT), [d]Consultation Care Measure (CCM), [e]Doctors' Interpersonal Skills Questionnaire (DISQ), [f]Experience of Service Questionnaire (ESQ), [g]Health Care Communication Questionnaire (HCCQ), [h]Instrument on Doctor-Patient Communication Skills (IDPCS), [i]Jefferson Scale of Patient Perceptions of Physician Empathy (JSSSPE), [j]The long term conditions questionnaire (LTCQ-6), [k]Matched Pair instrument (MPI) (PATIENT VERSION), [l]Norwegian Patient Experience Questionnaire (NORPEQ), [m]Oncology Patients' Perceptions of the Quality of Nusing Care Scale (OPPQNCS), [n]Patient Assessment of Care for Chronic Conditions (PACIC), [o]Patient-Centred Inpatient Scale (P-CIS), [p]Patient Experience with Inpatient Care (I-PAHC), [q]Patient Experience Questionnaire (Steine), [r]Patient Feedback on Consultation Skills (PFC), [s]Patient Participation in Rehabilitation Questionnaire, [t]Modified Perceived Involvement in Care Scale (M-PICS), [u]Person-centred Climate Questionnaire (PCCQ-patient version), [v]Picker Patient Experience Questionnaire (PPE-15), [w]Treatment-related empowerment scale (TES), [x]Intermediate Care for Older People Home-Based PREM (IC-PREM-Home), [y]Intermediate Care for Older People Bed-Based PREM (IC-PREM-Bed), [z]4 habits patient questionnaire (4HPQ), [aa]Patient assessment of integrated elderly care (PAIEC), [ab]Quality from the Patient's Perspective Questionnaire Shortened version (QPP-SF), [ac]Quality of End-of-life Communication Scale 10-Item Version (QOC-10), [ad]Relational and Management Continuity of Care (RMCC), [ae]Self-efficacy Scale for Chronic disease 6 item scale (SES6G), [af]Session Rating Scale (SRS), [ag]Shared Decision Making Questionnaire (SDM-Q-9), [ah]Scale to measure Therapeutic Relationship - Patient version (STAR-P), [ai]Views on Inpatient Care (VOICE), [aj]Quality of End-of-life Communication Scale revised (QOC-revised), [ak]Care evaluation Scale - Patient version (CES-P), [al]Components of Primary Care Index (CPCI), [am]Decision Self-Efficacy Scale (DSES), [an]Decisional Conflict Scale (DCS), [ao]Patient Approach and Views toward Healthcare Communication Scale (PAV-COM), [ap]Patient Perception of Continuity Instrument (PC), [aq]Control Preferences Scale (CPS), [ar]Health Confidence Score (HCS), [as]patient activation measure (PAM-13), [at]Person centred coordinated experience questionnaire (P3CEQ), [au]The Outcomes and Experience Questionnaire (OEQ), [av]Perceptions of care (POC), [aw]Palliative Care Outcome Scale (POS), [ax]Patient Experience Questionnaire (NHS survey), [ay]The Oxford Patient Involvement and Experience Questionnaire (OxPIE), [az]Quality of end of life care (QEOLC - 10), [ba]Health care system hassles scale (HCSHS), [bb]The Health Care Empowerment Questionnaire (HCEQ), [bc]Client Centred Care Questionnaire (CCCQ)
